# Supplementary material for: Implementing HLA-B*58:01 testing prior to allopurinol initiation in Malaysian primary care setting: A qualitative study from doctors’ and patients’ perspective
Source: PLoS One. 2024 Jan 11;19(1):e0296498. doi: 10.1371/journal.pone.0296498 (PMC10783771; doi:10.1371/journal.pone.0296498)
Supplement: S1 File — (DOCX) [file pone.0296498.s001.docx]

**Interview guide for primary care doctor**

For primary care doctors, the considerations are the issues with implementing HLA-B*5801 testing in their clinics and what factors influenced their decisions. Theoretical Domain Framework (doctor’s factor, organizational factor, etc) will be used as the conceptual framework to explore feasibility of implementing HLA-B*5801 testing from the perspective of primary care doctors.

| Domain |  |
| --- | --- |
| Knowledge | What do you understand about the use of testing? HLA-B*5801  What do you think about implementing HLA-B*5801 testing at primary care clinic? |
| Skills | What skills do you think are required to implement HLA-B*5801 testing at primary care clinic? |
| Beliefs about capabilities | Do you think you and your organisation (primary care clinic) are capable to carry out HLA-B*5801 testing at primary care clinic?  Are you confident? |
| Beliefs about consequences | Do you have any concerns of implementing HLA-B*5801 testing at primary care clinic?  Do you think this will benefit your patients? |
| Motivation and goals | Do you think you will sustain doing HLA-B*5801 testing at primary care clinic?  If yes, why? If no, why? |
| Environmental context and resources | What resources or training do you think you require before implementing HLA-B*5801 testing at primary care clinic? |
| Social influences | What information would you need to support the implementation of HLA-B*5801 testing at primary care clinic?  Would you involve patient in the structure or action plan? |
| Behavioural regulation | What action plan would you take before, during and after the implementation of HLA-B*5801 testing at primary care clinic?  Do you foresee any challenges? How do you intend to tackle? |
|  | |
|  |  |
|  |  |
|  |  |
|  |  |
|  |  |
|  |  |
|  |  |
|  |  |
