# Supplementary material for: Implementing HLA-B*58:01 testing prior to allopurinol initiation in Malaysian primary care setting: A qualitative study from doctors’ and patients’ perspective
Source: PLoS One. 2024 Jan 11;19(1):e0296498. doi: 10.1371/journal.pone.0296498 (PMC10783771; doi:10.1371/journal.pone.0296498)
Supplement: S2 File — (DOCX) [file pone.0296498.s002.docx]

**Interview guide for patients with gout**

**Preamble:**

We would like to ask you about what you think about a genetic test to identify a gene called HLA-B*5801. People with this gene are at risk for a severe reaction to allopurinol, which is the most common drug for treating gout.

**Topics**

Decisional conflict

- Would you be willing to take this test to see if you might be at risk of allopurinol reaction? [topic]
- How you feel about doing a genetic test in a primary care setting? [setting/ location]. Would you prefer to do it in a hospital or other healthcare facility (e.g. health lab)?
- No test is 100% accurate in predicting that patients will have a drug reaction even if they have the gene. Will you be willing to take the test, even if having the gene will not guarantee that you will have a drug reaction? Why or why not?

Knowledge and expectations/ Decision type timing, stage and leaning

- What do you know about this test?
- What are the pros of having this test?
- What are the cons of having this test?
- What is acceptable for you for the test in terms of
  - Procedure (method of collecting of genetic samples)
  - Waiting time for results (how long willing to wait at clinic for the results)
  - Cost of test (willingness to pay)

Values/ Personal/clinical characteristics

- Do you have any personal beliefs about genetic testing?
- How do you usually make treatment decisions about gout with your doctor? [shared decision making]

Support and resources

- What information would you like to have before taking an HLA-B*5801 test?
- What support would you like to have before taking a genetic test?
  - healthcare professional
  - family

**Ending**

- We are about to end the interview. Are there any other things you would like to mention regarding this test?
- Thank you.
