# Supplementary material for: Implementing HLA-B*58:01 testing prior to allopurinol initiation in Malaysian primary care setting: A qualitative study from doctors’ and patients’ perspective
Source: PLoS One. 2024 Jan 11;19(1):e0296498. doi: 10.1371/journal.pone.0296498 (PMC10783771; doi:10.1371/journal.pone.0296498)
Supplement: Attachment — Submitted filename: Response to reviewers.docx [file pone.0296498.s003.docx]

**Replies to reviewers**

**Manuscript number: PONE-D-23-27384**

Dear Editor and reviewers,

Thank you for giving us the opportunity to submit a revised draft of my manuscript titled “Implementing HLA-B*58:01 testing prior to allopurinol initiation in Malaysian primary care setting: A qualitative study from doctors' and patients' perspective”. We appreciate the time and effort that you and the reviewer have dedicated to providing your valuable feedback on my manuscript. We have made some changes to our manuscript to address the points raised by our reviewer. We have highlighted the changes within the manuscript.

Here is a point-by-point response to the reviewer’s comments.

**(the page and line number mentioned below are based on the tracked changes version of the manuscript)**

**Comment:**

I have some comments that relate to the limitations of this work.

One of the major issues stems from the fact that neither the clinicians nor the patients were familiar with the test. Hence, their opinions and perceived value of such a test would not be based on direct knowledge or data, and it would simply be based on supposition and assumptions. It would have been far more helpful to give them an information sheet that briefly summarises the characteristics of the test, including baseline probability of harm, positive and negative predictive values and number needed to test to prevent one serious adverse reaction. This method would have enabled capture of opinions based on genuine fact, rather than anybody's guess, particularly if the participants may have wildly differing ideas about the predictive values of such a test. In such a situation, it would have been very helpful to capture the participants perception of the predictive values and to assess if this is associated with heterogeneity in the opinion.

**Reply:**

We acknowledge this limitation which would affect the interpretation of our findings. While we did provide information regarding HLA-B*58:01 testing to our participants prior to the interviews, we did not include detailed information such as the baseline risk and accuracy of the test.

We have added a statement in Methods to inform regarding the provision of some basic information to our participants:

“Information regarding the risk of SCAR with allopurinol, association of HLA-B*58:01 allele with allopurinol-induced SCAR and the role of HLA-B*58:01 testing to prevent allopurinol-induced SCAR were included in the participant information sheet, The participants were allowed to clarify any queries on HLA-B*58:01 testing with the researchers at the beginning of the interviews.” (line 160-164, page 7-8)

We have also addressed this limitation in our Strength and limitations section:

“There were several considerations that may affect the interpretation of our findings. Firstly, most of our participants were not familiar with the test. The information on HLA-B*58:01 testing provided before the interviews did not include specific characteristics of the test such as the baseline risk, sensitivity and specificity of test, positive and negative predictive values and number needed to treat to prevent one allopurinol-induced SCAR, Hence their opinions and perceived value of the test may be based on their own supposition, which were not captured adequately in the study.” (line 589-595, page 26-27)

--------------------------------------------------------------------------------------------------------------------------------------

**Comment:**

It would help to know the average income in Malaysia, so that we can fully understand the context of the cost of such a test and treatments, as well as the need to pay for further clinic appointments at a private centre.

**Reply:**

We have included this information in the introduction:

“The median monthly income for employed citizens in Malaysia was RM2400 (USD 518) with an average monthly household income of RM8479 (USD 1831) in 2022.” (line 103-105, page 5)

--------------------------------------------------------------------------------------------------------------------------------------

**Comment:**

I'm surprised that none of the interviews were conducted in Chinese given that substantial numbers of the patients were of Chinese ethnicity. It seems to me therefore that the patient sample likely consisted of more highly educated people who were fluent in two or more languages. I feel that this qualitative study lacks generalizability because of this particular sample, and the lack of Indian ethnicity.

**Reply:**

The reviewer is correct in pointing this out as our Chinese patients were able to converse well in either English or Malay. While it is not unusual for the Chinese population in Malaysia to converse in two or more languages (Chinese in various dialects, English and Malay), we acknowledge that our patient sample consisted of more highly educated people. All our patient participants had at least secondary school education level with 10 of them with university degree. We took note of this and had addressed that the health literacy of our participants were likely higher in line 598, page 27.

We had revised our sentences in Strength and Limitations section to note the limited generalizability of our findings:

“Second, our findings may not be generalizable to all populations due to several factors. Our study was carried out in several health centres and clinics in Klang Valley, an urban area in Malaysia where health services are readily accessible to the population and the health literacy of our subjects may be higher. The issues raised in this study may differ from the participants in a rural setting. The ethnic diversity in our samples is limited by the absence of any Indian patients, the third largest ethnic group in Malaysia.” (line 595-600, page 27)

--------------------------------------------------------------------------------------------------------------------------------------

**Comment:**

There is a sentence in the limitation section where it is claimed that the sample from the Klang Valley would not differ substantially from the rural population. I don't think that this statement is supported by evidence, and my personal experience is that rural patients have very different ideas and expectations as compared to the large urban areas.

**Reply:**

We have rephrased our sentence in the Strength and Limitations section to acknowledge that our findings may differ from those in the rural population:

“The issues raised in this study may differ from the participants in a rural setting.” (line 598-599, page 27)

In addition to the above comments, we have also made some corrections to the spelling and grammatical errors.

We look forward to hearing from you regarding our submission and to respond to any further questions and comments you may have.

Sincerely,

Wei Leik Ng
